# Applying cognitive interviewing process to refine an instrument for measuring deprescribing experience and outcomes in older veterans

**DOI:** 10.1186/s41687-026-01145-7

**Published:** 2026-07-13

**Authors:** Helen Omuya, Sura AlMahasis, Ejura Salihu, Lauren Welch, Trisha Raňola, Justine Manning, Shiying Mai, Betty Chewning

**Affiliations:** 1 University of Alabama at Birmingham, Birmingham, USA; 2 University of Wisconsin–Madison, Madison, USA; 3 William S. Middleton Memorial Veterans Hospital, Madison, USA; 4 VA Geriatrics Research, Education and Clinical Center, Madison, USA

**Keywords:** Cognitive interviews, Older adults, Survey development, Measurements

## Abstract

**Background:**

Currently, there is no validated instrument that assesses a patient’s reported deprescribing experiences and outcomes. This study reports the third phase of refining such an instrument, the Deprescribing Evaluation and Quality Improvement instrument (DEQI), using cognitive interviews. Cognitive interviews have been used in the development of many survey tools; however, there is limited evidence of their use in pharmacy research, particularly in deprescribing studies involving older adults. The aim of this phase of the DEQI instrument development is to evaluate and refine the individual items to enhance their clarity to accurately reflect patient-reported experiences and outcomes after deprescribing interventions.

**Methods:**

A draft DEQI Instrument was tested through two rounds of cognitive interviews conducted four weeks apart, involving 16 Veteran adults aged 65 or older who were on multiple medications. Interviews lasted about 60–90 min. Two cognitive interviewing techniques, “think-aloud” and “verbal probing,” were used. Data from the interviews were independently analyzed by four coders employing two coding techniques: interaction coding and the cognitive classification scheme (CCS) to identify problematic questions in the DEQI instrument. Based on the analysis of the first round, the items were revised and then administered again in a second round. The improvement in clarity was evaluated.

**Results:**

Participants were mostly male (88%) and non-Hispanic White (69%), with a mean age of 73.4 years (range 64–84). About 38% had some college or technical school education. The classification coding scheme identified 102 problematic items in the first round, with approximately 70% related to the comprehension and communication code, and around 14% related to response selection. After modifications and readministration in the second round, the number of identified problems dropped significantly from 102 to 21. Following the revision in the second round, the proportion of “immediately codable” answers increased significantly (from 0.68 to 0.80), while the proportions of “uncodable” answers and “items requiring clarification” decreased.

**Conclusions:**

This study demonstrates the use of interaction coding and CCS to refine an effective evaluation instrument to improve deprescribing interventions with an older Veteran population. Through iterative refinement of items based on cognitive interviews, over half of the DEQI instrument’s items were improved, resulting in clearer items and reduced confusion among participants.

**Clinical trial number:**

Not applicable.

## Keypoint

This study demonstrates the use of cognitive interviewing, interaction coding, and the classification coding scheme to refine the deprescribing evaluation and quality improvement (DEQI) instrument.

Through iterative refinement during the cognitive interview process, older adults enhanced the clarity of items in the DEQI.

This involvement of older adults contradicts the idea that they would be unwilling to participate in or endure a lengthy interview.

## Background

A cognitive interview is a semi-qualitative research method that originated in the field of psychology and evolved from the Cognitive Aspects of Survey Methodology [1]. Cognitive interviews are useful for testing the clarity and effectiveness of questionnaires as tools for eliciting appropriate responses from participants. By critically evaluating how information in the questionnaire is perceived and interpreted by participants, cognitive interviews help researchers identify challenges with questionnaire administration (i.e., vague questions [2, 3], complex instructions, awkward syntax) that may confuse participants [4]. Cognitive interviews are also used to study how targeted audiences understand, mentally process, and respond to the survey questions with a special emphasis on potential breakdowns in this process [4]. 

According to Tourangeau (1984) and Willis (2004), participants go through four stages when responding to a question: (1) comprehend the question, (2) retrieve the information from memory, (3) decide how to respond and (4) select an answer from among the response categories that matches their internally generated answer [4, 5]. 

Cognitive interviews can be used to achieve six critical components: [6] (i) identify confusing questions and improve clarity, (ii) identify problematic questions or questions that are difficult to answer, (iii) ensure appropriate response options, (iv) assess whether questions are producing the intended data, (v) identify problematic question order and ensure logical order of questions, and (vi) reveal the thought process of participants on domain items. The application of cognitive interviewing to questionnaires helps identify inappropriate response scales, comprehension problems, question misinterpretation, and redundant questions, creating additional opportunities for item reduction [7]. When combined with expert review and subsequent pilot testing, cognitive interviews lead to a more robust instrument [4]. 

Cognitive interviews have been employed in the development of several survey instruments, such as the culturally adapted Illness Perception Questionnaire for African Americans, the Patient Reported Outcomes Measurement Information System (PROMIS), the Diabetes Symptom Self Care Inventory (DSSCI), the Patient’s Qualitative Assessment of Treatment – Real-World (PQAT-RW), and the Chronic Hypersensitivity Pneumonitis health-related quality of life (CHP-HRQOL) [2, 3, 8–11]. Despite the widespread use of cognitive interviews, there is a paucity of data on their application in pharmacy research, particularly in the context of deprescribing interventions. Deprescribing is the supervised, systematic withdrawal of medications for which the potential harms outweigh the benefits [12]. Currently, there is no validated instrument to assess deprescribing experiences and outcomes after the intervention. This highlights the need for a patient-centered instrument to measure patient-reported experiences and outcomes following their deprescribing interventions. To develop an instrument that clearly communicates questions about deprescribing experiences and elicits the intended responses, the items in the instrument need to be cognitively tested with the target populations. The goal is to help ensure that survey responses are not compromised by misunderstandings or misinterpretations.

This study is the third phase in the development of a Deprescribing Evaluation and Quality Improvement (DEQI) Instrument within the Veterans Affairs (VA) Healthcare System, where the VIONE (Vital, Important, Optional, Not indicated, Every medication should have an indication) medication optimization strategy is used for deprescribing interventions [13, 14]. The purpose of the DEQI instrument is to measure patient-reported experience and patient-reported outcome commonly referred to as ‘PREM’ and ‘PROM’ after a deprescribing intervention. The DEQI has five main constructs: (1) Changes in your medication which had items regarding the types of changes made to their medications, (2) Medications discontinued which had 32 items about their deprescribing experience and the outcomes of deprescribing, (3) Medications and perceptions which has 14 items about their knowledge and challenges they may have had with their medications, (4) Patient-Provider communication with 3 items, and (5) Getting medication through the VA system with also 3 items about the VA system. The first phase of the DEQI development involved qualitative interviews with Veterans to elicit information on their experiences and outcomes of the deprescribing intervention [15]. The second phase involved assessing items generated from the interviews by a Delphi panel of deprescribing experts. Using the insights from these interviews, the DEQI instrument was developed. The goal of the current study, this third phase, is to conduct cognitive testing of the DEQI instrument items with the target population and to modify them for future psychometric evaluation. The DEQI instrument will be innovative in its ability to capture PREM and PROM for deprescribing interventions.

**Objective**

The aim of this study is to refine items in the DEQI instrument through cognitive interviews and to improve their clarity.

## Methods

### Design of cognitive interviews for DEQI instrument

**Fig. 1:**
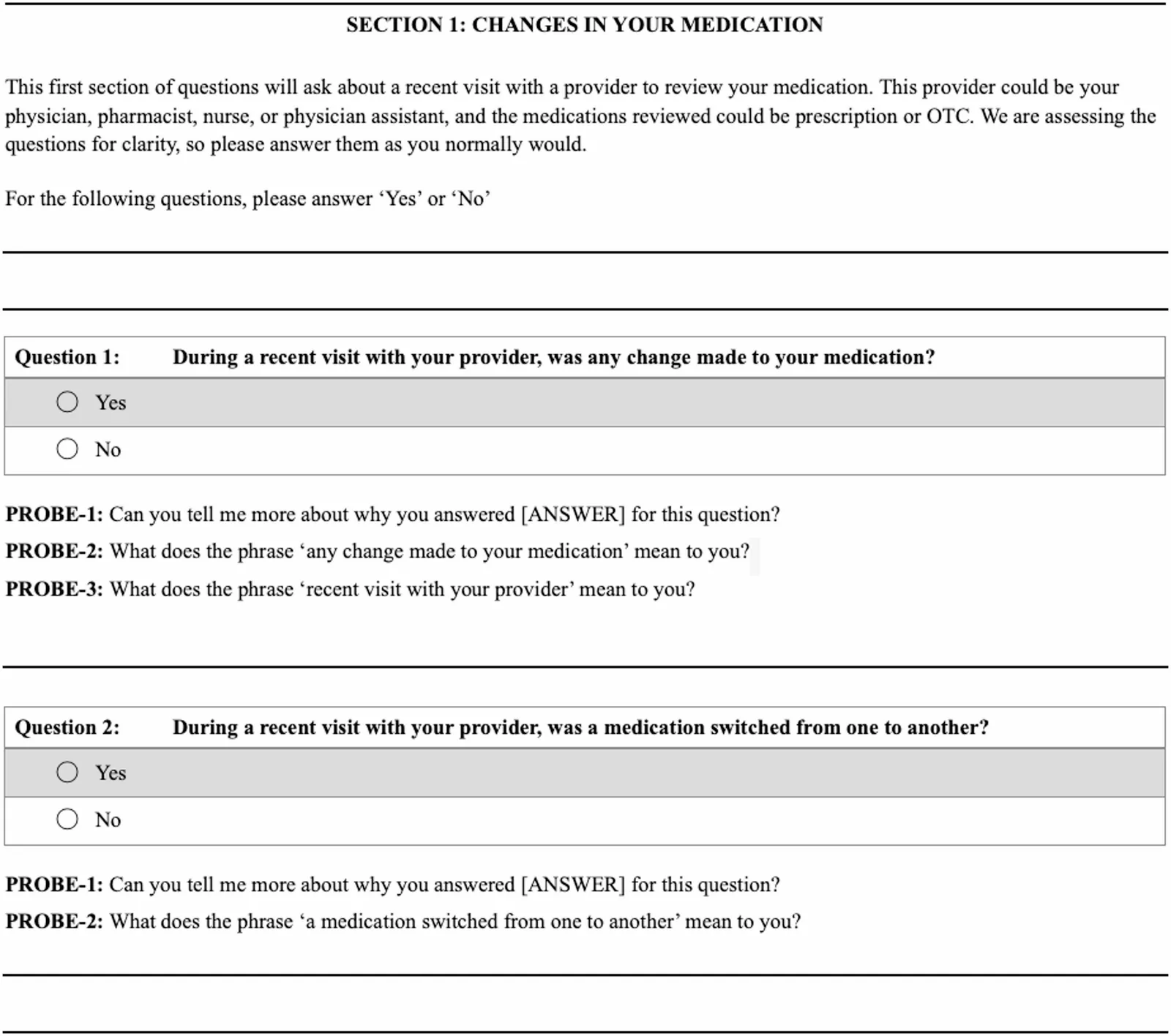
Interview guide for the interviewer

The draft DEQI Instrument items developed, modified, and validated through the Delphi process by deprescribing experts were converted into questionnaires that can be administered in person and virtually. The DEQI instrument tested included 58 items. The interviewer’s version of the questionnaire included both questions and their corresponding probes (See Fig. 1). For in-person interviews, a participant’s paper copy of the survey was created in booklet form, with one question per page. For virtual interviews, participants were shown a PowerPoint slide that contained individual questions on separate slides. In both formats, participants viewed only one question at a time, while the interviewer read and asked them the question verbally. This allowed participants to maintain focus on one question at a time. The participant’s copy of the questions did not contain the probes.

The items were tested in two rounds of cognitive interviews to assess the clarity of instructions, items, and response scales, as well as the ease of completion [6, 16, 17]. Two cognitive interviewing techniques were incorporated: the think-aloud technique and verbal probing [4, 18]. These were used to understand the participants’ cognitive processes as they responded to the questions. For the think-aloud approach, participants were asked and encouraged to vocalize their thoughts throughout the interview. Structured verbal probes were written for each question. The probes used were a combination of comprehension/interpretation, paraphrasing, recall, specific, and general probes [4]. Probes were drafted based on an analysis of likely problems the question might pose for one or more stages of the question-answer process. Although specific probes were developed for each question, spontaneous probes were allowed to ensure flexibility in response to the participant’s answer [19]. The cognitive interview guide included a draft of the questions to be administered in the survey.

### Study site and participants

The study took place in a VA healthcare system with 8 clinics serving Veterans from 19 rural and urban counties. The inclusion criteria for the study were Veterans aged 65 and older, taking 5 or more prescription medications daily, able to provide informed consent, and willing to answer survey questions about their medications for approximately 90 min. Veterans are individuals who served in the active military, naval, air, or space service and were discharged under conditions other than dishonorable [20]. Veterans who receive care from the VA are older and sicker than the Veteran population overall [21]. Participants were informed about the study through clinicians, word of mouth, and flyers. The flyer was also shared on a Veterans volunteer website. The flyer provided information on how to contact the study team if they were interested in the studyVeterans who were unable to speak English were excluded from the study. Participants were not screened on whether they had a deprescribing experience. Recruitment ended after the target of 16 Veterans was enrolled. The target sample size was determined based on prior literature studies and expert opinion [4, 10, 11]. No enrolled participant refused to participate in the study. An incentive of $100 was offered, comparable to that given in other cognitive studies [22, 23]. 

### Data collection

Interviews were conducted individually. In-person interviews took place at the VA site, while virtual interviews were conducted on the VA Microsoft Teams platform. Only the interviewer and participant were present during each interview. Before the initial interview, the questionnaire was pre-tested with a standardized patient to assess the flow of questions and probes. The survey was modified accordingly. Two rounds of cognitive interviews were conducted, with eight Veteran participants in each. Depending on the respondent’s preference, interviews were conducted either virtually or in person. At the start of the interview, the interviewer, a female pharmacist and Ph.D. candidate trained in qualitative research and survey design, orally explained the purpose of the interview and the analysis techniques [4]. Survey items were presented to the participants, who were asked to verbalize the mental process involved in providing their answers using a combination of think-aloud and probing techniques [4, 6, 17]. After they responded to the survey item, probing questions were administered to better understand their cognitive process in answering the question. These scripted, structured probes were asked after each question, based on the respondent’s answers. Examples of these probes are (1) Can you tell me more about why you answered [ANSWER] for this question? (2) In your own words, can you tell me what this question means to you? (3) How easy was it for you to answer? How did you come up with this answer [ANSWER] [9]? During the survey, when participants indicated confusion or uncertainty, the interviewer elicited their feedback on how to phrase the item more clearly.

The purpose of the interviews was to identify problematic questions, not to achieve data saturation. The interview duration ranged from 37 to 90 min, with an average of 70 min. In the first round, a version of the survey was administered to eight participants. The items were modified based on the analysis from this initial round. In the second round, two versions of the survey were administered and tested, following common practice to assess improvements [24]. The different versions had wordings for six items to determine which was clearer for the Veteran. The interviewer took notes during the interview, and depending on their responses, item modifications sometimes occurred after an interview, as suggested by Willis (2005) [4]. No relationship was established between the interviewer and participants before the study, and participants had no personal knowledge of the interviewer. The interviewer did not provide any personal views to the participants before the interview.

### Data analysis

The interview data were transcribed, and the analysis used a combination of quantitative and qualitative approaches. The quantitative approach tallied the frequencies of participants’ interpretations of the questions or difficulties encountered and rated each participant’s understanding as adequate or inadequate, while the qualitative approach identified patterns of problems or recurrent, problematic themes among participants [4]. A coding scheme employing two techniques, interaction coding and formal analysis using the cognitive classification coding scheme (CCS) developed by Rothgeb et al. (2001), was used to code the transcripts generated for each respondent for potential problems with the questions using the coding scheme [6, 24–27]. 

The coding scheme was created using Microsoft Excel. Each transcript was independently coded by two analysts, with a total of four analysts. To ensure rigor in the analytic process and minimize the impact of subjective bias, the interviewer (H.O.) did not participate in the coding but was available to clarify context whenever disagreements arose. The improvement in item clarity after re-administration was compared using a t-test of means to assess the significance of the difference between rounds.

#### Interaction coding

Also referred to as behavior coding, interaction coding was employed to evaluate each item in the interview transcripts. The unit of coding was at the Exchange Level, which is considered an adjacency pair of a question and an answer, and typically is the first two exchanges [27]. Each item was analyzed based on eight codes: (i) immediately codable answer, (ii) implicitly codable answer, (iii) codable answer + qualification, (iv) codable answer + elaboration, (v) uncodable answer, (vi) seeks clarification, (vii) interruption, and (viii) don’t know or refusal [26]. These codes assess participants’ cognitive processing of survey questions, identify problematic survey interviewer behaviors, and analyze the interactions between the interviewer and the respondent [25, 26]. 

#### Formal analysis of items with probes

Selective coding using the CCS was employed as a second method to identify five problem categories based on 28 codes [4, 5, 28, 29]. Four broad categories of problems that are identifiable with the CCS are: (i) Comprehension and Communication, which reflects the encoding process, (ii) Memory Retrieval, which captures the recall process, (iii) Judgment and Evaluation, which reflects the judgment process, and (iv) Response Selection for the response process. Any problem that could not be categorized in these categories was placed in an ‘other’ group.

The coding pair of analysts met with the interviewer to discuss and resolve all discrepancies, ensuring consensus.

## Results

The developed questionnaires were used in cognitive interviews conducted in two rounds, with eight Veterans per round (*N* = 16). A total of 18 Veterans were screened, and two were excluded as recruitment became selective to ensure the sample reflected the educational levels of the target population. The mean age of participants was 73.4 years (range 64 to 84). Most participants were male (87.5%) and non-Hispanic White (68.8%). See Table 1 for participants’ descriptions.

**Table 1:** Demographic description of Veterans interviewed

Characteristic	*N* (%)
**Gender**	
Male	14 (87.5)
Female	2 (12.5)
**Race/Ethnicity**	
Non-Hispanic White	11 (68.8)
Non-Hispanic Black	4 (25)
Asian	1 (6.2)
Hispanic	0 (0)
**Age**	
Min	64
Mean	73.4
Max	84
**Education**	
High school diploma or GED	3 (19)
Some college or technical school	6 (37.5)
College or technical school graduate	1 (6)
Post-college or graduate degree	6 (37.5)

GED = General Educational Development

### Interaction coding

The proportion of items with responses across the interaction code categories in both rounds is shown in Table 2. The total number of interaction codes (*n*) applied varied between the two rounds, as some items were skipped when not applicable. For example, some participants who had not restarted or discontinued a medication were not required to provide codable responses to these questions. Participants provided a high proportion of immediately codable answers in both rounds. However, as shown in Table 2, after the items were modified in the second round, there was a statistically significant improvement in the proportion of immediately codable answers (t (780) =-3.703, *p* < 0.001) and a decrease in the proportion of uncodable answers (t(780) = 3.387, *p* < 0.001) and items requiring clarification (t(780) = 2.298, *p* = 0.011). Although the proportions of items with implicitly codable answers, codable answers with elaborations, and codable answers with qualifications decreased in the second round of interviews, these reductions were not significant. Furthermore, there was also no difference in the proportion of items coded as ‘don’t know or refusal’. In both rounds, the participants did not appear to interrupt the interviewer while the main question was being read.

As interaction coding was only applied to the first adjacent pair of exchanges between the interviewer and the participants about a single item, the responses they initially chose were not necessarily their final answers, as they sometimes changed their answers after the probing questions were asked. Additionally, when they gave an answer that seemed to contradict a previous response, the interviewer would repeat the survey item for clarification. In these cases, the respondent appeared to misunderstand the question initially and provided a more consistent response.

**Table 2:** Proportion of items using interaction coding from round 1 & 2*

Description	Round 1	Round 2
Immediately Codable Answer	**0.679**	**0.801**
Implicitly Codable Answer	0.031	0.023
Codable Answer + Qualification	0.013	0.005
Codable Answer + Elaboration	0.144	0.119
Uncodable Answer	**0.086**	**0.028**
Seeks Clarification	**0.034**	**0.013**
Interruption	0.000	0.000
Don’t Know or Refusal	0.013	0.013
*n*	383	399

^*^Bolded numbers represent significant difference between the proportions in both rounds from a two-tailed t-test, *p* < 0.05, *n* = total number of coded applied

### Problems identified using the classification coding scheme

A total of 102 problems were identified across 58 items during the first round of cognitive interviews. Among these, 34 (58.6%) were revised. Approximately 70% were related to comprehension and communication, while about 14% were related to response selection. Participants’ feedback was carefully considered in the item revisions.

After revising and re-administering items in a second round of cognitive interviews to a different set of 8 Veterans, the number of identified problems decreased from 102 to 21. Details on the kinds of problems identified are described below and summarized in Table 3.

**Table 3:** Identified problems based on the CCS System

Problem Label	Round 1			Round 2	
	Frequency	%	Frequency		%
**Comprehension and Communication**					
**Question Content**					
Complicated instruction	7	6.9%	0		0.0%
Vague topic/term	25	24.5%	7		33.3%
Complex topic	4	3.9%	0		0.0%
Topic carried over from earlier question	3	2.9%	0		0.0%
Undefined term(s)	11	10.8%	0		0.0%
**Question Structure**					
Complex, awkward syntax	6	5.9%	3		14.3%
Erroneous assumption	8	7.8%	5		23.8%
**Reference Period**					
Undefined	7	6.9%	3		14.3%
**Memory Retrieval**					
High detail required or information unavailable	6	5.9%	0		0.0%
**Judgment and Evaluation**					
Complex estimation	9	8.8%	1		4.8%
**Response Selection**					
**Response Terminology**					
Undefined term(s)	2	2.0%	0		0.0%
Vague term(s)	3	2.9%	0		0.0%
**Response Structure**					
Missing categories	9	8.8%	0		0.0%
**Other**	2	2.0%	2		9.5%
**Total**	**102**	100.0%	**21**		100.0%

#### A. Comprehension and communication

Problems in the process of how the participants understood the questions included challenges with the question content, structure, and reference period. As stated earlier, this category represented the majority of the identified problems in the Round 1 interviews.

#### Question content

In this category, all four types of problems described by the CCS were identified - vague topic/term, complex topic, topic carried over from earlier questions, and undefined terms. The majority of problems were related to the use of vague terms (25%). Participants had difficulties with the use and context of some terms and phrases in the questions, such as ‘recent visit’, ‘uncomfortable’, and ‘saw no reason to stop’. They also felt that some words in the questions were different from those they usually use when communicating with their provider.

Undefined terms accounted for about 11% of the overall problems. Participants asked for clarification of terms such as ‘number of medications.’ They also wondered whether taking medication twice a day counted as one or two medications. Additionally, when two medications were discontinued, some participants shared that they had difficulty answering the questions as they had different experiences with each of them, although for them, the most challenging experience stood out in their memory. They also revealed that when thinking of their providers, they do not usually consider their pharmacist as a provider. Regarding a question about plans to follow up after a deprescribing intervention, participants expressed that the use of the word ‘plan’ with ‘follow up’ had different meanings to them. They shared that their follow-ups were not necessarily planned. They wondered what constituted as a plan to follow up, whether this needed to be an in-person visit, or if a call from their provider would suffice. The two questions below show how one respondent understood these terms in the Round 1 interview.

Par 4 Q8

I: After meeting with your provider, was there a plan to follow up on the changes made to your medication?

P: For this one, the word plan is helpful.

I: Okay.

P: So I’d say, if there’s a written plan, specifically stated plan for medication, I would say no. But you say it as follow up, so follow up could be a plan.

I: Okay.

P: So it says plan to follow up, but for me, the word plans don’t—I’ve always followed up appointments with the person who prescribes the medication, or at least contact. They have MyChart you can contact your provider so there’s follow up.

Par 4 Q9

I: Who made plans to follow up on the changes made to your medication, you or your provider?

P: Can I answer both?

I: Yes.

….

P: … Most of my healthcare providers …. sometimes they’ll say check back with me. And sometimes I’ll just say, I’ll check back with you.

I: Okay.

P: And here the word plan has a different meaning. It doesn’t throw me off on this one.

I: Okay.

P: Because a plan here can just be—just follow up on something.

I: Okay. But the first one does?

P: Yeah, the first word plan and the other one has a different meaning.

#### Question structure

Two types of problems were identified with the question structure. They were erroneous assumptions and complex/awkward syntax, which accounted for about 8% and 4% respectively of the problems in the first round of the interviews.

Regarding the comfort of stopping a medication, although a respondent in round 1 gave an immediately codable answer, on probing, the respondent shared that the question could be interpreted in two different ways.

Par 2 Q17

I: How uncomfortable were you with stopping the medication your provider asked you to stop?

P: Yesterday, maybe a little.

I: Okay. What does the word ‘uncomfortable’ mean to you?

P: That would be my discomfort with the decision making. I don’t think it would be—I don’t interpret it, in this case, as a result of the medication. Like I was on pain med, and I can’t have anymore, and then I really would be uncomfortable. I think that could—that word could be interpreted two different ways.

The respondent also had challenges with a question that asked about how taking a medication interfered with their daily activities. They wondered if this was referring to the act of swallowing the medication or to potential side effects of the medication.

Par 2 Q44

I: In the last three months, how often did taking your medication interfere with your day-to-day activities?

P: Extremely often.

I: Okay.

P: Not particularly taking it, the side effects of the medication. So that’s interesting. Yeah.

I: Okay.

P: The side effects of the meds.

I: Okay. Do you think this question needs to be better phrased? ‘Cause it’s not necessarily the taking it, it’s the effects of it?

P: It’s the effects of it. I think most people would go to the effects of it rather than the actual administration of it. Although, you know what, if you put somebody on meds more than once or twice a day, the taking of it turns into a problem too.

I: Yeah.

P: So I—you know, like somebody’s gotta give themselves insulin four times day that’s a huge, huge impingement. Even taking antibiotics, I mean, think of how many people don’t—because they miss doses because it’s three times a day instead of whatever. So I—maybe that does need to be clarified a little bit.

Several items regarded questions about a patient continuing a medication that the provider had instructed them to discontinue. The framing of these questions, *“Did you continue taking the medications because …”* was confusing to some participants as they wondered why they would continue a medication if they had been asked to stop.

Par 1 Q31

I: Did you continue taking the medication because you had a bad experience in the past after stopping a medication?

P: No.

I: Can you tell me what the phrase ‘bad experience in the past’ meant to you?

P: Taking a medication that caused adverse effects in the past. Why would you continue taking the medication?

I: Okay. Thank you. So looking at this question again, what does the phrase ‘after stopping a medication’ mean to you?

P: If a provider asked you or required you to stop a medication and you had an adverse effect because you stopped taking it, I would question why the provider made me stop taking it. You know, I don’t know. It’s—I’m not sure that’s a good question.

I: So could you tell me more about your answer?

P: Did you continue taking the medication because you maybe were afraid to have a bad experience from the medication you stopped taking in the past? I don’t know. Why would you continue taking a medication if you experienced bad things?

#### Reference period

In this category, the problem identified was undefined terms, accounting for 7% of overall problems. Participants who had discontinued several medications at various times were uncertain about how to answer the question about the effects of stopping a medication, since their experiences from the different instances of drugdiscontinuations had varied. More specificity was needed for these questions.

Par 2 Q 16

I: How often do you have any unwanted effects because of stopping the medication your provider asked you to stop; never, rarely, sometimes, very often, extremely often?

P: As far as stopping a med, I have not had an adverse effect.

I: …So it’s gonna be never, right?

P: I would say, never.

I: Okay. Can you tell me more about why you answered never for this question?

P: Well, you know, maybe I shouldn’t say never. Are you talking about just the medication from yesterday or the medications that I’ve taken over my illustrious career of being a patient?

#### B. Memory retrieval

The problem in this category was related to not having the required details or information to answer the question, accounting for approximately 6% of the identified problems in the first round of interviews. Although some participants answered the questions with codable answers, when probed further, they mentioned that they could not remember the details or that their medications interfered with their mental capacity, preventing them from recalling.

Par 5 Q 18

I: Okay. After the visit with your provider to discuss stopping the medication, how concerned were you about the number of medications you take; not at all, a little, somewhat concerned, very concerned, extremely concerned.

P: Okay. I’m not sure how to answer that because I’ve taken a lot of medications most of my life, my adult life, and stopping a medication doesn’t concern me at all. Like I say, I trust his judgment. In this particular case, that I can relate to, it was replaced with a more effective medication.

I: All right. So here we’re asking about the number of the medications you take.

P: Okay. Yeah. I’d be happier if I didn’t take all these medications, but I know why I’m taking them.

#### C. Judgment and evaluation

Problems with using information retrieved to make necessary judgments, as well as the selection and reporting of answers, were also noted among some participants, accounting for about 9% of identified problems. Although they indicated that they understood the intention behind the questions and provided codable answers, participants sometimes felt that there could be multiple ways to answer the questions.

Par 6 Q 46

I: How often are you afraid of possible, unwanted effects from your medication; never, rarely, sometimes, very often, extremely often?

P: Again physically, not really concerned. Mentally, extremely often.

I: Okay. If you have this question in front of you, what will you answer; never, rarely, sometimes, very often, extremely often?

P: I have to ask you to ask that again.

I: How often are you afraid of possible, unwanted affects from your medication?

P: Very often.

Par 4 Q 42

I: In the last three months, how often did you get an up-to-date list of your medication from your healthcare team: never, rarely, sometimes, very often, extremely often?

P: I’d say extremely often because on MyChart, I can get it anytime I want.

I: Beautiful. Thank you.

P: The way this is worded it says, from your healthcare team. So I’m not thinking—my healthcare team doesn’t send me my list. I get—they update the list on MyChart so, for me, I just go on and get it.

I: Okay. So maybe we rewrite this to how often do you have access—did you_.

P: List of your medications. I think you just say, how often do you get an up-to-date list of your medications. I don’t think you have to say who it’s from.

#### D. Response selection

When determining the appropriate response to a question, issues with missing response categories and terminology were identified, accounting for approximately 14% of the problems. Participants indicated that multiple options could apply to an item that required them to select only one. Conversely, some questions did not apply to certain participants, suggesting that a ‘not applicable’ option was needed. There were also instances where the respondent selected a response that was not listed, and the interviewer had to repeat the options.

Par 6 Q9

I: Who made plans to follow up on the changes made to your medication, you or your provider?

P: Made plans to—a combination. The two of us made the decision.

Par 3 Q 56

I: When a provider outside the VA gives you a prescription order, how often do you have a hard time getting the medication through the VA pharmacy; never, rarely, sometimes_.

P: Rarely, never. I mean they’re really good about—they actually sent me—I just got a text today telling me my medication was up for renewal. Do I wanna continue_.

I: Yeah.

P: _or stop it? There’s certain things where they were giving me too many so I said stop.

I: Okay. So this question is asking about a provider outside the VA.

P: Outside the VA?

I: Yes. When a provider outside the VA gives you a prescription order_.

P: I don’t.

I: You don’t have any provider outside_.

P: A 100% with the VA.

### Item modification based on feedback from first round of cognitive interviews

As described above, the problems identified in the first round included the sequence and order of questions, issues with comprehension, unclear definitions, intensely worded questions, a lack of reference period, ambiguity of some questions, and incomplete or unclear options in the response scales. Table 4 presents some initial items and their revised modifications for the second round of interviews based on feedback from the participants.

A. **Sequence and order of questions**: The order of questions was altered in several instances to prevent participants from preemptively answering a question that would be asked later. For example, regarding the changes in medication construct, some participants viewed switching from one medication to another as stopping one and adding a new one. However, the survey items distinguished these actions, and separate items were created for switching, adding, and stopping a medication. In this case, the sequence of the questions was adjusted to position the question about switching medication before the questions on adding or stopping medication, thereby improving comprehension of their differences.
B. **Providing clearer definitions**: Participants sometimes suggested defining specific terms, such as ‘provider’ and ‘specialist’, and clarifying whether medications included over-the-counter options. They also noted that some side effects of medications could be mental, which was not clear in the definition. Definitions were provided to clarify the meanings of these terms.
C. **Reducing ambiguity**: Participants noted that some questions could be interpreted differently than intended. For example, forgetting to take a medication and missing a dose are different actions; a missed dose might be intentional, whereas a forgotten dose is usually unintentional. In another case, the participant was unsure whether the question inquired about the medication’s effect or its administration. These items were revised to be more precise and specific.
D. **Rephrasing questions**: Participants suggested that some items be rephrased, wordings be changed, or that qualifiers be included. The incorporation of these suggestions improved the clarity of items to convey the purpose of a question effectively. In other cases, items were rephrased to demonstrate event precedent and facilitate easy comprehension.
E. **Using less intense words**: Participants suggested that some words were too intense and recommended using softer language. For example, some participants felt that *‘afraid’* and *‘worry’* were too intense and suggested that they be changed to *‘concerned’*. They also suggested that the phrase ‘unwanted effect’ should be replaced with the more common term ‘side effect’, as this is more understandable.
F. **Removing words or phrases**: In other cases, participants suggested deleting certain words because they might be confusing and may not apply to all situations referenced by the context in the question.
G. **Clearer Response categories**: Based on participants’ suggestions, the response categories were also modified. Participants noted that in some instances, all the response options were applicable. These questions were changed from a ‘*choose one*’ option to a *‘select all that apply’* option. In another instance, they suggested that a ‘*Not applicable*’ option should be included as the questions may not apply to everyone. Furthermore, some expressed that the time reference scale was not clear, and to improve clarity, the *‘sometimes’* option for frequency was changed to *‘occasionally’.* For another item, they suggested that the response scale needed to be time-bound, as the possibilities could be subjective and mean different things to different individuals. For this item, a period-bound reference category that specified duration in days/weeks was provided.

**Table 4:** Sample items and revisions made based on participant feedback

Modification Type	Initial item	Revised item
Rephrasing Questions	In the last three months, how often did ***taking*** your medication**(s)** interfere with your day-to-day activities?	In the last three months, how often did ***the effect of your*** medication interfere with your day-to-day activities?
	After ***your meeting with your provider***,*** was there a plan to*** follow up on the changes made to your medication?	After ***meeting with your provider***,*** was there a discussion on when*** to follow up on the changes made to your medication?
Removing word/phrases	How helpful would it be to have a follow-up ***visit*** after you stop taking a medication(s)?	How helpful would it be to have a follow-up after you stop taking a medication(s)?
	In the last three months, how often did you get an up-to-date list of your medications ***from your health care team***?	In the last three months, how often did you get an up-to-date list of your medications?
Reducing ambiguity	In the last three months, how often did you ***miss taking*** a dose of your medication as prescribed?	In the last three months, how often did you ***forget to take*** a dose of your medication as prescribed?
	In the last three months, how often did ***taking*** your medication(s) interfere with your day-to-day activities?	In the last three months, how often did ***the effect of*** your medication interfere with your day-to-day activities?
Less Intense Words	How often are you ***afraid*** of possible unwanted effects from your medication(s)?	How often are you ***concerned*** about any possible side effects from any of your medication(s)?
	How often do you ***worry*** about the cost of your medication(s)?	How *often* are you ***concerned*** about the cost of your medication(s)?
Clearer Response Category	In the last three months, how often did you ***miss taking*** a dose of your medication as prescribed? ◯ Never ◯ Rarely ◯ Occasionally ◯ Very often ◯ Extremely often	In the last three months, how often did you ***forget to take*** a dose of your medication as prescribed? ◯ Never ◯ Rarely***/Once a month*** ◯ Occasionally/***2–3 times a month*** ◯ Very often***/Once a week*** ◯ Extremely often***/More than once a week***

### Refinement summary

Cognitive interviews were used to make minor revisions to 34 out of 58 items in the instrument. After the first round, the wording of 31 items was rephrased, the response scales of 9 items were modified, and the sequence order of 5 items was changed. These modifications significantly improved the proportion of immediately codable answers and decreased the number of problems identified when the survey was readministered in the second round. In the second round, which involved two versions of six questions, it was clear after using one version in two interviews that it was very confusing. Therefore, the second version was used for subsequent interviews.

## Discussion

Cognitive interviews are used to identify problems in a survey and suggest ways to revise the items before full-scale data collection. They have been widely utilized in the development of several instruments.

For this study, cognitive interviews were used to achieve six critical goals: [6] (i) identify confusing questions and improve clarity, (ii) identify problematic questions or questions that are difficult to answer, (iii) ensure appropriate response options, (iv) assess whether questions are producing the intended data, (v) identify problematic question order and ensure logical order of questions, and (vi) reveal the thought process of participants on domain items. The application of cognitive interviewing to the questionnaire helped identify inappropriate response scales, comprehension problems, question misinterpretation, and redundant questions, creating additional opportunities for items reduction in the DEQI [7]. 

Two rounds of cognitive interviews were conducted with the target population, and the transcripts from these interviews were analyzed to identify and refine problematic questions. This ensured that the items in the survey were clear, understandable, and accurately measured the construct of interest when administered to the target population. Some insights from the interviews are shared below.

i. **Assessment of participants’ participation**: Cognitive interviews can be mentally demanding for participants, especially those in the older age groups. The use of probing techniques also increases the duration of the process. As noted by Garcia (2011), this could be a limitation of cognitive interviews [8]. Participants were informed that the interview could last up to 90 min. Although they had the option to take a break during the session, all participants chose to complete it in one sitting. Being transparent about the time required helped manage participants’ expectations. This endurance on the part of the participants contradicts the notion that older adults would not be willing to remain for such an extensive interview period.
ii. **Probing techniques and variations in initial and final answers**: During the first adjacent pair of exchanges between the interviewer and the respondent, a codable answer was usually obtained. However, when asked follow-up questions, participants sometimes changed their response choices, suggesting they might not have fully understood the question. This could indicate an issue with accuracy when surveys are administered without probes. When a survey is administered, a method to verify the accuracy of the respondent’s responses may be needed.
iii. **Assessment of interaction coding**: Following the revision of the items, there was a noticeable increase in the percentage of answers that could be immediately coded. During the interviews, participants were asked to think aloud by verbalizing their thoughts. This might explain why the proportion of codable answers with elaboration was high in both rounds and showed no significant change after item refinement in the second round. However, the participants’ explanations helped clarify why some questions could be unclear and how they could be improved. For this assessment, an interruption was defined as when the respondent cuts off the interviewer while the main question is being read. Instances where the respondent answered the question before hearing the response scale were not considered interruptions. This was because the respondent had a copy of the questions and could see and read them on their own. Using this description, no interruption was noted in both rounds.
iv. **Instrument Refinement from Cognitive Interviews**: In the first round of interviews, approximately 70% of the identified problems related to comprehension and communication. Problems involving vague or unclear terms and undefined terms accounted for over 50% of these problems. These findings align with previous research by Rothgeb (2007), who also found that these two problems dominated their qualitative coding results [30]. Analysis of the findings in this study led to minor revisions in over 50% of the items. These revisions mainly involved rephrasing an item, removing a word or phrase, rearranging the item sequence, defining specific terms, and clarifying response options. These revisions, following cognitive interviews, are consistent with a previous study by Morgan et al. (2014) [31]. The revisions to the draft DEQI instrument resulted in a significant reduction in the number of problems identified after the second round.

### Research implications

This study has several implications for future research. First, during the interviews, despite repeated prompts to think aloud, participants mostly did not verbalize their thought processes as they decided how to answer an item. However, after responding, some participants elaborated on why they answered the item as they did. This is consistent with several other studies, which found that think-aloud may not be as effective, and that probing techniques were better at exploring specific terms in each question [32]. The merits of the think-aloud technique need to be further assessed.

Furthermore, when interpreting interaction-coding results, the method of administering the interview must be taken into consideration. With the think-aloud technique, where participants are encouraged to verbalize their thoughts, this may lead to a high frequency of the ‘*codable answer with elaboration’* code and may not necessarily indicate a problem with the question. Further research is needed to determine the optimal coding scheme for various cognitive interviewing techniques.

### Limitations

This study has provided valuable insights into the cognitive interviewing process with an older adult population. However, some limitations in the process need to be taken into consideration. First, participants were not screened for prior experience with deprescribing; however, the interviews were designed to ensure that the questions were clear to the target population. Although some items did not apply to these participants with no deprescribing experience, they were still able to provide feedback on the clarity of the survey items.

Secondly, verbal tones and non-verbal cues could not be captured and analyzed from transcripts. Hence, comprehension challenges expressed non-verbally could not all be analyzed by simply reading the transcripts. Future studies should consider analyzing video recordings alongside interview transcripts to capture nuances that could be missed with transcripts alone. Finally, the cognitive interviews were conducted in the Veteran population from one study site. This may limit the generalizability of study findings to other settings.

## Conclusion

This study illustrates how interaction coding and CCS can be used to refine an effective evaluation tool for enhancing deprescribing interventions. Through iterative improvement of items based on cognitive interviews, more than half of the DEQI instrument’s items were improved, resulting in clearer questions and less confusion among participants. The next phase of this research involves conducting broader surveys for psychometric evaluation to establish the reliability and validity of the DEQI instrument. The findings from this work contribute to the growing evidence supporting patient-centered deprescribing by offering an instrument to assess patients’ reported experiences and outcome measures related to deprescribing interventions.

## Data Availability

The datasets used and/or analyzed during the current study are available from the corresponding author on reasonable request.
